# HBV Reactivation During the Treatment of Non-Hodgkin Lymphoma and Management Strategies

**DOI:** 10.3389/fonc.2021.685706

**Published:** 2021-07-01

**Authors:** Xing Cao, Yafei Wang, Panyun Li, Wei Huang, Xiaojuan Lu, Hongda Lu

**Affiliations:** ^1^ Department of Oncology, The Central Hospital of Wuhan, Tongji Medical College, Huazhong University of Science and Technology, Wuhan, China; ^2^ Department of Respiratory and Critical Care Medicine, The Central Hospital of Wuhan, Tongji Medical College, Huazhong University of Science and Technology, Wuhan, China

**Keywords:** hepatitis B virus reactivation (HBV-R), risk factors, antiviral prophylaxis, non-Hodgkin lymphoma (NHL), rituximab, immunosuppressive therapy

## Abstract

Hepatitis B virus reactivation (HBV-R), which can lead to HBV-related morbidity and mortality, is a common and well-known complication that occurs during the treatment of non-Hodgkin lymphoma (NHL) patients with current or past exposure to HBV infection. HBV-R is thought to be closely associated with chemotherapeutic or immunosuppressive therapies. However, immunosuppressive agents such as anti-CD20 antibodies (e.g., rituximab and ofatumumab), glucocorticoids, and hematopoietic stem cell transplantation (HSCT) administered to NHL patients during treatment can cause deep immunodepression and place them at high risk of HBV-R. In this review, we explore the current evidence, the guidelines of several national and international organizations, and the recommendations of expert panels relating to the definition, risk factors, screening and monitoring strategies, whether to use prophylaxis or pre-emptive therapy, and the optimal antiviral agent and duration of antiviral therapy for HBV-R.

## Introduction

Hepatitis B virus (HBV) is a double-stranded DNA virus belonging to the family Hepadnaviridae (1). HBV infection has reached epidemic proportions globally. It is estimated that over one-third of the world’s population has been infected with HBV, with approximately 248 million of them suffering from chronic infection (2, 3). Compared with uninfected individuals, those infected with HBV have a 2–3-fold greater risk of developing non-Hodgkin’s lymphoma (NHL), particularly diffuse large B-cell lymphoma (DLBCL), which represents the major NHL subtype (4–9). Although the mechanism underlying this phenomenon remains unclear, it is likely to be due to the hepatotropic and lymphotropic nature of HBV, which can assure HBV replication in lymphoid tissue (10–12). Interestingly, it has been reported that HBV infection is uncorrelated with Hodgkin’s lymphoma (HL) (13, 14). HBV reactivation (HBV-R) is defined as resolved/occult HBV that becomes active again, leading to adverse consequences. Occult HBV infection is characterized by the presence of replication-competent HBV DNA (i.e., covalently closed circular DNA [cccDNA] comprising the episomal HBV genome) in the liver and/or blood of hepatitis B surface antigen (HBsAg)-negative individuals as determined by currently available assays (15). Over recent years, a close link has been established between HBV-R and cytotoxic chemotherapeutic drugs, such as anthracyclines, cyclophosphamide, vincristine, and prednisone, as well as anti-CD20 monoclonal antibody therapy (e.g., rituximab) (16–21). Nevertheless, an R-CHOP regimen comprising rituximab, anthracyclines, cyclophosphamide, vincristine, and prednisone is now widely used as the first-line treatment for NHL, highlighting the need to be vigilant for HBV-R in concerned patients. The rate of HBV-R in HBsAg(+) patients who undergo rituximab-containing therapy is reported to range from 33 to 65% and between 6 and 24% in those negative for HBsAg and positive for anti-hepatitis B core antibody (HBcAb), respectively (22). The clinical manifestations of HBV-R can vary from asymptomatic hepatitis to lethal liver failure (23). Additionally, patients with HBV-R may postpone scheduled chemotherapy or present with abnormal liver function, leading to adverse effects on treatment outcome for the primary disease (24, 25). There are no standard screening, monitoring, or management strategies for HBV-R, and recommendations for the clinical management of HBV-R for patients treated with R-CHOP differ among institutions. Here, we review the recent literature relating to HBV-R, as the appropriate and timely identification of HBV-R, as well as suitable strategies for its management, remain crucial for improving the quality of life of NHL patients.

## Definition of HBV-R

No uniform criteria for the definition of HBV-R currently exist and different guidelines have heterogeneous definitions for HBV-R diagnosis and management. The American Association for the Study of Liver Diseases (AASLD), the Asian Pacific Association for the Study of the Liver (APASL), and the American Gastroenterological Association (AGA) give clear definitions of HBV-R (26–28), whereas the European Association for the Study of the Liver (EASL) does not (29). **Table 1** summarizes the definitions of HBV-R based on the different guidelines. Additionally, the AASLD also provides explicit concepts for HBV-associated hepatitis, namely, acute serum alanine aminotransferase (ALT) levels ≥3-fold higher than that at baseline and an absolute value >100 U/L (26). Interestingly, it has been reported that monitoring ALT levels can lead to the earlier detection of HBV-R because ALT levels increase 2–3 weeks before a rise in HBV DNA levels is detected (31).

**Table 1:** Definition of HBV-R according to different guidelines.

Guidelines	Reactivation of chronic infection	Reactivation of resolved infection
American Association for the Study of Liver Diseases. (AASLD, 2018) (26)	One of the following: 1) a ≥100-fold elevation in the HBV DNA load compared with the baseline level; 2) ≥1,000 IU/ml of HBV DNA with an undetectable baseline level; 3) ≥10,000 IU/ml HBV DNA if the baseline level is not available.	One of the following: 1) HBV DNA is detectable; 2) reappearance of HBsAg
The Asian Pacific Association for the Study of the Liver. (APASL, 2016) (27)	One of the following: 1) a ≥100-fold increase in the HBV DNA load from baseline levels; 2) the reappearance of HBV DNA to a level of 100 IU/ml if the baseline level is undetectable; 3) ≥20,000 IU/ml HBV DNA if the baseline level is not available.	One of the following: 1) reverse HBsAg seroconversion (reappearance of HBsAg); 2) the appearance of HBV DNA in serum if HBsAg is negative
American Gastroenterological Association. (AGA, 2015) (30)	One of the following: 1) *de novo* detectable HBV DNA if baseline level is undetectable; 2) a ≥10-fold increase in HBV DNA levels if the baseline DNA level is detectable.	A change in HBsAg status (negative to positive).
European Association for the Study of the Liver (EASL, 2017) (29)	Not defined	Not defined

HBsAg, hepatitis B surface antigen; HBcAb, anti-hepatitis B core antibody.

Chronic infection: HBsAg(+) and HBcAb(+) patients.

Resolved infection: HBsAg(−) and HBcAb(+) patients.

Collectively, HBV-R can be identified in a timely manner by combining ALT levels with HBV DNA and HBsAg. In HBsAg(+) patients, a dramatic rise in HBV DNA concentrations (usually 100-fold or more) are indicative of HBV-R, while in patients with resolved HBV infection (HBsAg[−] and HBcAb[+]), HBV-R usually means the reappearance of HBsAg or an increase in serum HBV DNA concentrations with or without HBsAg seroconversion and ALT exacerbation.

## Risk Factors and Possible Mechanism of HBV-R During NHL Treatment

Notably, HBV-R can be induced by coinfection with HBV and severe acute respiratory syndrome coronavirus 2 (SARS-CoV-2) (32–34). Other coinfections, such as that with HCV or HDV, can also increase the likelihood of HBV-R (35). The crucial risk factors for HBV-R can be classified into three types, namely, host-related, virus-related, and medication-related (35). Medication-related factors are usually associated with underlying disease that requires immunosuppressive therapy, such as chemotherapy, solid organ or bone marrow transplantation, rheumatological conditions, dermatological conditions, or inflammatory bowel disease) (36). As mentioned above, the drugs that are currently used to treat NHL (anthracyclines, cyclophosphamide, vincristine, prednisone, and anti-CD20 monoclonal antibody) are usually immunosuppressive, and constitute the focus of this review.

### Host-Related Risk Factors

Males are more prone to undergoing HBV-R than females. Yeo et al. reported that in 600 HBsAg(+) cancer patients exposed to chemotherapy, the HBV-R ratio was almost three-fold higher in men than women (37, 38). Additionally, people that are more than 50 years old, HBeAg(+), and with underlying disease that requires immunosuppressive therapy (e.g. lymphomas) are at greater risk of developing HBV-R (37).

### Virus-Related Risk Factors

The virus-related, high-risk factors for HBV-R identified to date include detectable HBV DNA, HBsAg and HBcAb positivity, mutations in HBsAg, and HBV genotype (23, 39). Among these, detectable HBV DNA was reported to be the most important predictive factor for HBV-R. In one study, 37.8% of patients (31/82) with detectable viral load developed HBV-R (*P* = 0.0003, odds ratio [OR] 8.4, 95% CI 2.6–27.2) (39). Salpini et al. further identified mutations in HBsAg as being risk factors for HBV-R. The authors analyzed HBsAg-associated genetic features and found that 75.9% of patients (22/29) with HBV-R carried HBsAg mutations compared with 3.1% for control patients (2/64; *P* < 0.001). Among the HBsAg mutations identified, 61.5% were found to reside in a major hydrophilic region, while some were known immune escape-associated mutations, such as the sD144E mutation that disrupts humoral response-mediated HBsAg recognition. The remaining mutations were found to reside in class-I/II-restricted T-cell epitopes, suggesting that they were important for HBV escape from T-cell-mediated responses (40). HBsAg(+) patients have an eight-fold higher likelihood of HBV-R when compared with patients with resolved infection (HBsAg[−] and HBcAb[+]) (41). Notably, HBV genotype A is rarely involved in HBV-R, while other genotypes are reported (35, 42). Coinfection with other viruses, such as HCV, HDV, HIV, or SARS-CoV-2, also puts patients at higher risk of HBV-R, as mentioned before (26, 35).

### Medication-Related Risk Factors

Numerous studies have indicated that immunosuppressive and chemotherapeutic medications represent the major risk for HBV-R (43–45). The greatest and most reported risk for HBV-R is associated with B-cell-depleting therapy, such as that with the anti-CD20 antibody, rituximab (35). The AGA and the AASLD have stratified the HBV-R-related risk of individual medications or therapies (26, 30). Additionally, the risk of HBV-R has been graded through different immunosuppressive treatments and HBV infection status, as follows (**Table 2**): (1) Very high risk, greater than 20% chance of reactivation, and is associated with anti-CD20 antibody therapy (rituximab or ofatumumab) and hematopoietic stem cell transplantation (HSCT); (2) high risk, between 10 and 20% chance of reactivation, and is mainly related to high-dose glucocorticoid and anthracycline treatment; (3) moderate risk, between 1 and 10% chance of reactivation; (4) low risk, less than 1% chance of reactivation (30, 35, 45–47). Importantly, the immunosuppressive therapies included in the very-high- and high-risk groups are those usually administered to NHL patients, rendering them prone to HBV-R and incidental adverse events.

**Table 2:** HBV-R risk groups based on patient infection status and associated immunosuppressive treatment.

Risk group	HBV-R rate (%)	Hepatitis status	Associated medications
Very high risk	>20%	Chronic infection	Anti-CD20 monoclonal antibodies (rituximab, ofatumumab), HSCT
High risk	10–20%	Chronic infection	Anthracycline derivatives: daunorubicin, doxorubicin, epirubicin High-dose glucocorticoids (>20 mg/day for 4 weeks and longer) Anti-CD52 antibody: alemtuzumab
Moderate risk	1–10%	Chronic infection	Cytotoxic therapy without glucocorticoids: cyclophosphamide, vincristine; TNF-α inhibitors: infliximab, etanercept, golimumab, adalimumab; Cytokine and integrin inhibitors (mogamulizumab); Tyrosine kinase inhibitors (TKIs): imatinib, dasatinib, nilotinib; Proteasome inhibitors: carfilzomib
Low risk	<1%	Chronic infection	Glucocorticoids (methotrexate or azathioprine) lasting less than a week or a low-dose (<10 mg prednisone) within 4 weeks
		Resolved infection	High-dose glucocorticoid or the anti-CD52 antibody alemtuzumab

HBsAg, hepatitis B surface antigen; HBcAb, anti-hepatitis B core antibody; HSCT, hematopoietic stem cell transplantation; TNF, tumor necrosis factor.

Chronic infection: HBsAg(+) and HBcAb(+) patients.

Resolved infection: HBsAg(−) and HBcAb(+) patients.

#### Anti-CD20 Antibodies—Rituximab and Ofatumumab

Rituximab, approved in 1997, is used for the treatment of NHL and chronic lymphocytic leukemia. Numerous cases of HBsAg(+) lymphoma patients treated with rituximab-containing therapy developing HBV-R have been documented since 1999 (48, 49). Rituximab and ofatumumab are humanized anti-CD20 monoclonal antibodies targeting CD20, a cell-surface marker on B lymphocytes, resulting in B-cell depletion and the subsequent impairment of B-cell antigen-presenting function, with the consequent reduction of specific anti-HBV CD4-positive T-cell activation and proliferation (50). Rituximab affects cell signaling by directly inducing the apoptosis of malignant B cells and activating complement-dependent cytotoxicity. These effects result in the rapid death of rituximab-targeted cells and the activation of natural killer (NK) cells, which then produce interferon-gamma (IFNγ), and thereby induce antibody-dependent cellular cytotoxicity when they interact with rituximab-coated target cells (51).

Ofatumumab functions through a similar mechanism. These types of drugs are commonly associated with serious HBV-R-related events and can increase the risk of hepatocyte dysfunction and mortality if HBV-R is not quickly identified and cleared (35, 52). Few retrospective data are available for the incidence of HBV-R in HBsAg(+) patients treated with rituximab-containing therapy without any antiviral prophylaxis. The HBV-R rate in NHL patients has been reported to vary between 18.2 and 80%. HBV-R occurs from after a few weeks to up to 55 months after rituximab administration, indicating that rituximab represents a very high-risk factor for HBV-R (48, 53–57). Between 1997 and 2009, the US Food and Drug Administration (FDA) MedWatch Database reported 118 cases of HBV-R. Among these patients, those administered rituximab-containing therapy had a prominently increased risk of HBV-R compared with those given non-rituximab-containing therapy (OR 5.73, 95% CI 2.01–16.33, *P* = 0.0009) without heterogeneity (19). Consequently, the FDA alerted healthcare professionals that rituximab and ofatumumab were associated with a high risk of HBV-R and added “boxed warnings” (the strongest warnings) to the product labels in September 2013 (58). In the Emerging Trends Conference sponsored by the AASLD held in 2015, entitled “Reactivation of Hepatitis B,” HBV-R was reported to be a possible underestimated clinical challenge related to ofatumumab or rituximab treatment. Furthermore, it was suggested that all patients undergoing ofatumumab- or rituximab-containing therapies should be screened for HBV-R, and that HBsAg(+) or HBcAb(+) patients should start prophylactic antiviral therapy to prevent HBV-R (59).

Collectively, these data indicate that rituximab and other B-cell-depleting therapies pose the greatest risk for HBV-R (35, 46, 60), which warrants vigilance by the medical community.

#### Corticosteroids

Corticosteroids were first reported to be associated with HBV-R by Sagnelli et al. in 1980, which was subsequently widely confirmed (38, 61). The risk for HBV-R with corticosteroid use is deemed to be dose- and time-dependent, with studies having indicated that a high dose (>20 mg/day) of chronic prednisone therapy for longer than 4 weeks is associated with a high risk of HBV-R (28, 62). Cheng et al. conducted a randomized study on 50 HBsAg(+) lymphoma patients and compared the HBV-R rate in patients undergoing an identical chemotherapeutic regimen receiving or not corticosteroid treatment. The authors reported that the cumulative incidence of HBV-R at 9 months was significantly higher in the corticosteroid treatment group (38 *vs*. 73%, *P* = 0.03) (63). In a recent 6-year prospective cohort study, HBV-R was reported to occur a median of 10 months (range, 4–32) after steroid administration (53).

Corticosteroids enhance HBV replication mainly through two mechanisms. First, they suppress cell-mediated immunity *via* the inhibition of interleukins, which then prevents T and B cell proliferation (43). Second, corticosteroids stimulate the glucocorticoid-responsive element present in the HBV genome, thereby exerting a direct suppressive effect on T-cell-mediated immunity (23, 64). Corticosteroids were also reported to be able to increase HBsAg secretion *via* inducing autophagy, i.e., inhibiting autophagy using 3-MA, an autophagy inhibitor, decreased HBV replication and HBsAg secretion (65); however, this possibility requires further investigation. These observations suggest that the reported HBV-R in patients coinfected with SARS-CoV-2 and HBV might be associated with corticosteroid use during the treatment of COVID-19. The R-CHOP regimen, which contains prednisone, is still conventionally administered to patients with NHL as first-line standard therapy. Hence, the risk for HBV-R should be taken into consideration when treating NHL patients with a corticosteroid-containing regimen.

#### Anthracyclines

Anthracyclines, such as doxorubicin and epirubicin, have also been linked to a high risk of HBV-R (35, 66). This class of chemotherapeutic drugs is applied to treat a variety of solid and hematological cancers, including lymphoma, bladder cancer, soft-tissue sarcoma, leukemia, breast cancer, and multiple myeloma. Doxorubicin exerts its HBV-R-related effects by increasing the expression of the cell cycle regulator p21 (Waf1/Cip1), which upregulates the expression level of enhancer-binding protein α (C/EBPα) and, consequently, promotes the binding of C/EBPα to the HBV promoter; this, in turn, enhances HBV transcription, and thus also viral replication (67). Nevertheless, it is difficult to evaluate the anthracycline-associated risks for HBV-R as these drugs are often used in combination with other immunosuppressive or chemotherapeutic agents, such as rituximab.

#### Immune Checkpoint Inhibitors

Immune checkpoint inhibitors (ICPIs), such as antibodies targeting programmed cell death protein 1/programmed cell death 1 ligand 1 (PD-1/PD- L1) (anti-PD-1: pembrolizumab and nivolumab; anti-PD-L1: atezolizumab, durvalumab, and avelumab) and cytotoxic T-lymphocyte-associated protein 4 (anti-CTLA4: ipilimumab and tremelimumab), have been used as immunotherapy in the treatment of various types of cancer. Pembrolizumab is an FDA-approved agent used to treat relapsed or refractory primary mediastinal large B-cell lymphoma after ≥2 prior lines of therapy. It has been suggested that this class of drugs may give rise to HBV-R. Given that their mechanism of action involves activating the immune system, it seems unlikely that ICPIs induce HBV-R (35); however, sporadic incidents of HBV-R after ICPI administration have been described in case reports and a retrospective cohort study (68–72). In the latter, of 114 HBsAg(+) patients given anti-PD-1/PD-L1 antibody therapy, six (5.3%) developed HBV-R in a median time of 18 weeks after treatment initiation (72). Therefore, given the concern that ICPI treatment may lead to HBV-R, prophylactic antiviral therapy should be an appropriate option for HBV-infected or resolved patients undergoing ICPI therapy.

Other biological agents, such as TNF-α inhibitors (infliximab, etanercept, golimumab, and adalimumab), tyrosine kinase inhibitors (TKIs: imatinib, dasatinib, and nilotinib), the Janus kinase (JAK) 1/2 inhibitor ruxolitinib, and the proteasome inhibitor bortezomib, are also thought to be related to HBV-R; however, they are not currently applied to lymphomas (38, 44).

## Screening Before Treatment

The prevention of HBV-R begins with patient screening. Numerous approaches have been adopted by different organizations and institutions to address the issue of screening for HBV infection before the initiation of immunosuppressive therapy. There is a consensus among various cancer governing bodies that all patients at high risk of HBV-R or those receiving B-cell-depleting therapies should be screened before the initiation of therapy (26, 30, 35). The AGA, AASLD, EASL, and the American Society of Clinical Oncology (ASCO) screening guidelines are presented in **Table 3** (26, 28, 29, 73). The National Comprehensive Cancer Network (NCCN) Guidelines for B-cell lymphomas suggest that both HBsAg and HBcAb should be tested among NHL patients before the beginning of immunosuppressive treatment, especially that involving anti-CD20 antibody-containing regimens. The baseline HBV DNA burden should be obtained if patients are positive for HBsAg or HBcAb to quickly detect HBV-R and take the appropriate measures (74).

**Table 3:** Guidelines for the screening, management, and monitoring of HBV-R during immunosuppressive therapy.

Guidelines	Screen object	Management strategies for HBV-R	Duration of antiviral therapy	Monitoring during prophylaxis	Monitoring after prophylaxis
American Association for the Study of Liver Diseases (AASLD, 2018) (26)	All patients	Chronic infection: prophylaxis Resolved infection: either prophylaxis or pre-emptive therapy, but prophylaxis is needed when patients are receiving anti-CD20 antibody therapy	At least 6 months (12 months for patients receiving anti-CD20 antibody therapy) after the completion of immunosuppressive therapy	Not mentioned	HBV DNA levels should be tested every 1–3 months
The Asian Pacific Association for the Study of the Liver (APASL, 2016) (27)	All patients	HBsAg(+) cancer patients: prophylaxis; Resolved HBV with detectable HBV DNA: prophylaxis; Resolved HBV with undetectable HBV DNA: pre-emptive therapy, except with anti-CD20 antibody therapy or stem cell transplantation	At least 12 months after the cessation of therapy	Resolved HBV with undetectable HBV DNA: ALT and HBV DNA every 1–3 months	Not mentioned
American Gastroenterological Association (AGA, 2015) (30)	Patients at moderate or high risk of HBV-R	High and moderate risk: prophylaxis; Low risk: routine prophylaxis not recommended	The same as for AASLD	No recommendation	No recommendation
European Association for the Study of the Liver (EASL, 2017) (29)	All patients	HBsAg(+) patients: prophylaxis; Resolved HBV, high risk: prophylaxis; Resolved HBV, moderate and low risk: pre-emptive therapy (monitor HBsAg and HBV DNA every 1–3 months)	At least 12 months after the cessation of immunosuppressive treatment and at least 18 months for rituximab-based regimens	Not mentioned	Liver function tests and HBV DNA should be tested every 3–6 months
American Society of Clinical Oncology (ASCO, 2020) (73)	All cancer patients	HBsAg(+) patients: prophylaxis; Resolved HBV, high risk: prophylaxis; Resolved HBV, moderate and low risk: pre-emptive therapy (monitor HBsAg and HBV DNA every 3 months)	For a minimum of 12 months following anticancer therapy	ALT and HBV DNA levels should be tested every 6 months during antiviral therapy	HBsAg(+) and resolved HBV with high risk: at least monthly for the first 3 months after the cessation of antiviral therapy and every 3 months thereafter

HBsAg, hepatitis B surface antigen; ALT, alanine aminotransferase.

Owing to the immunosuppressive agents used in the treatment of NHL that place patients at a very high, high, or moderate risk of HBV-R, all NHL patients should be tested for HBV infection (at least for HBsAg and HBcAb) before the initiation of therapy to minimize the risk of HBV-R and related complications, including mortality.

## Management Strategies for HBV-R in Clinical Practice

After screening, the next challenge is the management of HBV-R in individuals receiving immunosuppressive agents. There are two strategies targeting HBV-R, namely, antiviral prophylaxis and pre-emptive therapy. Antiviral prophylaxis means treating patients (usually at least 1 week before immunosuppressive therapy) with HBsAg(+) or HBcAb(+) regardless of viral load or whether or not there are clinical symptoms of HBV-R. Pre-emptive therapy refers to the close surveillance of HBV DNA, in which antiviral therapy begins at the first sign of an increase in the HBV DNA load (26, 74, 75). The guidelines for management strategies are also presented in **Table 3**.

In oncological practice, it is usually too late to take measures to deal with HBV-R and schedule treatments are interrupted when HBV-R begins to take shape causing poor outcomes for NHL patients. Consequently, we think that it is appropriate to initiate antiviral prophylaxis before immunosuppressive therapies are given to NHL patients.

### Agents for Antiviral Prophylaxis

To date, no antiviral medication has been approved for the prevention of HBV-R. Two types of treatment are available for patients with chronic HBV infection, including nucleoside analogs (NAs) and interferon-alpha. However, interferons are no longer conventionally used to treat lymphoma patients owing to the associated intolerance, adverse effects, and selective effectiveness (46). Hence, several NAs, such as entecavir (ETV), lamivudine (LMV), adefovir, and tenofovir, are the only effective options available for the treatment of HBV-R among NHL patients.

#### Lamivudine

LMV is a cost-effective alternative for HBV treatment and was the first NA approved for this purpose. Consequently, it has been widely used for antiviral prophylaxis among NHL patients (76). In the first study, none of 30 HBsAg(+) patients with lymphoma undergoing intensive chemotherapy experienced HBV-R in the LMV prophylaxis group, whereas 8 out of 15 patients (53%) in the no-prophylaxis arm had HBV-R (*P* = 0.002). Survival free from HBV-R-related hepatitis in the group receiving LMV prophylaxis was significantly longer than that of the control group (*P* = 0.002 on the log-rank test) (77). In the second randomized trial, among 51 patients undergoing CHOP chemotherapy, HBV-R was detected in 30.8% (95% CI, 14.3–51.8%) of those undergoing LMV prophylaxis *vs*. 60% (95% CI, 38.7–78.9%) for those not receiving LMV treatment (*P* = 0.05) (78). A meta-analysis involving 16 studies reported that the HBV-R rate was significantly lower in patients receiving LMV prophylaxis than in those of the control group (8.6% [11/127] *vs*. 50.6% [136/269], respectively), suggesting that LMV can reduce the incidence of HBV-R (relative risk [RR] 0.21, 95% CI 0.13–0.35), as well as HBV-related mortality (RR 0.68, 95% CI 0.19–2.49) (79). A recent retrospective study (80) on consecutively enrolled HBsAg(−) and HBcAb(+) NHL patients who received rituximab-based chemotherapy found that none of the patients who were given LMV prophylaxis experienced HBV-R or treatment-related side effects (81). However, the long-term use of LMV is liable to generate a high rate of drug resistance, especially when used beyond 1 year. The incidence of resistance to LMV has been reported to be as high as 20% in patients treated for more than 1 year with non-immunosuppressive-containing medication (30); this incidence escalated dramatically to 30% after 2 years, and then increased exponentially with continued use (76). The most commonly identified mutation conferring resistance to LMV occurs in the tyrosine–methionine–aspartate–aspartate (YMDD) motif of the HBV-DNA polymerase gene (82). Hence, owing to the low threshold for the generation of resistance to LMV (83), this drug was replaced by next-generation NAs such as ETV or tenofovir that possess a high barrier to resistance, as demonstrated by multiple studies and meta-analyses (28, 80, 84, 85).

#### Entecavir and Tenofovir

ETV and tenofovir disoproxil fumarate are new-generation NAs that have a high barrier to drug resistance and superior viral suppressive capability (23, 86–88). A retrospective analysis of HBsAg(+) NHL patients (stage III–IV) who received antiviral prophylaxis with ETV (*n* = 34) or LMV (*n* = 89) during chemotherapy suggested that ETV-treated patients had lower rates of hepatitis (5.9 *vs*. 27.0%, *P* = 0.007) and HBV-R (0 *vs*. 12.4%, *P* = 0.024), as well as fewer interruptions of chemotherapy (5.9 *vs*. 20.2%, *P* = 0.042) (89) compared with those treated with LMV. A prospective, randomized, multicenter clinical trial conducted in China that included 121 patients compared the efficacy of ETV (*n* = 61) and LMV (*n* = 60) in preventing HBV-R among HBsAg(+) patients undergoing R-CHOP treatment for DLBCL. The results indicated that patients in the ETV group had a markedly lower incidence of HBV-related hepatitis (0 *vs*. 13.3%, *P* = 0.003) and HBV-R (6.6 *vs*. 30%, *P* = 0.001), as well as fewer interruptions of chemotherapy (1.6 *vs*. 18.3%, *P* = 0.002), with respect to those in the LMV group. No difference in terms of incidence of adverse events was identified between these two agents (90). Another meta-analysis that included 770 lymphoma patients also confirmed that patients with HBsAg(+) receiving LMV prophylaxis during chemotherapy had a significantly higher chance of HBV-R compared with those receiving ETV (OR 5.0, *P* < 0.001) (80). In a study conducted to evaluate the effectiveness of tenofovir for the treatment of HBV-R in patients undergoing immunosuppressive treatment, 25 of 38 patients were given tenofovir as prophylaxis, and 13 were administered the drug as pre-emptive therapy. None of the patients receiving tenofovir as prophylaxis developed HBV-R during immunosuppression. In addition, the remaining 13 patients received tenofovir at the first sign of HBV-R and all had a complete biochemical and virological response within 9 months (91).

Based on the above observations, ETV or tenofovir (especially ETV) is recommended as the standard agent for the prevention and treatment of HBV-R by NCCN, AASLD, and others as they possess superior viral suppressive ability and a high barrier to resistance (26, 35, 74, 92).

### Duration of Therapy and Monitoring

The optimal duration of prophylactic antiviral therapy remains controversial. Data derived from multiple sources indicate that antiviral prophylaxis should last for at least 6 months after the cessation of immunosuppressive therapy, and should be lengthened to 12 months for patients receiving regimens with B-cell-depleting therapies or antiviral prophylaxis (46, 86). The reason for extending the duration of antiviral prophylaxis use is that immune recovery may take longer, while the immunosuppressive effects of rituximab have been reported to persist for longer than 1 year after the last delivery (35, 93). HBV-R has also been reported to occur more than 2 years after the completion of rituximab-containing chemotherapy (54, 94). The AGA, AASLD, EASL, and ASCO guidelines regarding the duration of therapy and monitoring are presented in **Table 3**. Additionally, the NCCN guidelines for B-cell lymphomas recommend that monitoring and antiviral prophylaxis should be continued for at least 12 months after the completion of anticancer treatment, and that HBV DNA levels should be tested monthly during treatment, and then every 3 months after completion of antiviral prophylaxis (74).

In the clinic, prophylactic antiviral therapy tends to be delivered before or at the onset of anticancer therapy to patients with prior HBV infection, regardless of baseline HBV DNA levels, because HBV-R has been reported to occur even after 1 year of infection. Consequently, close monitoring and longer use of antiviral prophylaxis should be considered, particularly in patients receiving anti-CD20-antibody-containing therapy (84, 95–97).

## Discussion

HBV-R in NHL patients undergoing immunosuppressive therapies, especially rituximab-containing treatment, is now a well-recognized and preventable complication in clinical practice. Nevertheless, there is a large knowledge gap in our understanding of this disease process, making HBV-R a vexing and persistent problem. Among NHL patients with chronic HBV infection, HBV-R has gained extensive attention because of the associated significant morbidity and mortality. The immunosuppressive therapies used during the entire NHL treatment period, such as anti-CD20 antibody therapy, glucocorticoid treatment, and HSCT, usually amplify the odds of HBV-R, as mentioned before. Whether to screen patients at risk, stratify patients for risk based on HBV serological status and type of immunosuppression, whether to use prophylaxis or pre-emptive therapy, and the identification of the optimal antiviral agent and treatment duration remain unresolved issues.

Several screening strategies have been proposed by different organizations, namely, risk factor-based, risk-adaptive, and universal screening. HBV testing rates in cancer patients before therapy based on risk factors have been estimated to be low (19–55%) (98–100); however, the morbidity associated with HBV risk factors among patients with cancer may be high (101). Universal screening is the preferred option for the AGA and the AASLD given the limitations of risk factor-based and risk-adaptive screening. These two organizations recommend universal HBV screening as a reasonable and cost-effective strategy before anticancer therapies are administered to reduce the risk of HBV-R (26, 30). Accordingly, it is appropriate to test HBsAg, HBcAb, and HBsAb (if available) among all NHL patients to identify and cure HBV-R earlier and provide better clinical outcomes.

The current guidelines agree that HBsAg(+) patients or those receiving anti-CD20 antibody therapy or HSCT are at high risk of HBV-R and should be given prophylaxis until after the cessation of anticancer therapy. Among moderate or low-risk HBV-R patients, pre-emptive antiviral therapy initiating at the first sign of HBV-R may also be a decent option. Recent studies and multiple meta-analysis have demonstrated the greater efficacy and lesser drug resistance of ETV and tenofovir as first-line agents for the prevention of HBV-R. The optimal duration of prophylaxis for HBV-R remains unclear. Indeed, Tasuku et al. reported a rare case of a 54-year-old woman diagnosed with DLBCL and HBsAg(−) and HBcAb(+) in whom HBV-R occurred 55 months after the completion of chemotherapy (54). Owing to the deep immunodepression exerted by rituximab or HSCT, lifelong antiviral treatment may be the better option (23, 102).

In the era of immunotherapy, numerous novel agents have emerged targeting the treatment of NHL and other solid cancers; however, prospective data showing how they interact with the immune system and their relation to HBV-R are limited. Additional, well-designed, prospective studies are needed to allow the stratification of patients at risk of HBV-R as well as a better understanding of the appropriate antiviral therapy and the optimal duration of prophylaxis. With accumulating evidence and experience, it is expected that HBV-R can be avoided, identified, controlled, and, perhaps cured.

## Author Contributions

HL conceived and designed the study and reviewed the manuscript. XC and YW collected the data and wrote the manuscript. PL, WH, and XL revised the manuscript. XC designed and wrote the table. All authors contributed to the article and approved the submitted version.

## Conflict of Interest

The authors declare that the research was conducted in the absence of any commercial or financial relationships that could be construed as a potential conflict of interest.
